# Metabolomic analysis of *Trichophyton rubrum* and *Microsporum canis* during keratin degradation

**DOI:** 10.1038/s41598-021-83632-z

**Published:** 2021-02-17

**Authors:** Anita Ciesielska, Anna Kawa, Katarzyna Kanarek, Adrian Soboń, Rafał Szewczyk

**Affiliations:** 1 Department of Molecular Microbiology, Faculty of Biology and Environmental Protection, University of Lodz, Lodz, Poland; 2 LabExperts sp. z o.o., Gdańsk, Poland

**Keywords:** Fungal pathogenesis, Metabolomics, Skin diseases, Microbiology, Fungi

## Abstract

Keratin is important and needed for the growth of dermatophytes in the host tissue. In turn, the ability to invade keratinised tissues is defined as a pivotal virulence attribute of this group of medically important fungi. The host–dermatophyte interaction is accompanied by an adaptation of fungal metabolism that allows them to adhere to the host tissue as well as utilize the available nutrients necessary for their survival and growth. Dermatophyte infections pose a significant epidemiological and clinical problem. *Trichophyton rubrum* is the most common anthropophilic dermatophyte worldwide and its typical infection areas include skin of hands or feet and nail plate. In turn, *Microsporum canis* is a zoophilic pathogen, and mostly well known for ringworm in pets, it is also known to infect humans. The aim of the study was to compare the intracellular metabolite content in the *T. rubrum* and *M. canis* during keratin degradation using liquid chromatography system coupled with tandem mass spectrometer (LC-MS/MS). The metabolite “fingerprints” revealed compounds associated with amino acids metabolism, carbohydrate metabolism related to the glycolysis and the tricarboxylic acid cycle (TCA), as well as nucleotide and energy metabolism. The metabolites such as kynurenic acid, l-alanine and cysteine in case of *T. rubrum* as well as cysteine and riboflavin in case of *M. canis* were detected only during keratin degradation what may suggest that these compounds may play a key role in the interactions of *T. rubrum* and *M. canis* with the host tissue. The metabolomic results were completed by qPCR gene expression assay. Our findings suggest that metabolomic analysis of *T. rubrum* and *M. canis* growing in culture media that mimic the dermatophyte infection could allow the understanding of processes involved in the pathogenesis of dermatophytes.

## Introduction

Dermatophytes such as *Trichophyton rubrum* and *Microsporum canis* belong to a group of filamentous fungi infecting keratinised structures, including the skin, hair, and nails. *T. rubrum* is representative of anthropophilic fungi and responsible for over 60% of all dermatophytosis such as *Tinea pedis*, *Tinea corporis*, *Tinea inguinalis*, *Tinea unguium* as well as deep dermal infections^1^. *M. canis* is one of the main etiological agents of dermatophytosis in animal mainly cats and dogs. However, this zoophilic dermatophyte can be transmitted to humans through direct contact with infected animal via arthrospores^1^. A keratinolytic activity and pathogenesis of this group of medically important fungi is corelated because, during infection, dermatophytes secrete a lot of proteases that degrade keratinised structures into oligopeptides as well as free amino acids, which are then used by the fungi as sources of nutrients^2^. Proteolytic degradation of keratin is possible after relaxation of its structure by reduction of disulphide bridges (S–S). Sulphite [SO_3_^2−^] is excreted by the sulphite efflux pump SSU1, and acts as a reducing agent. The loosened structure of the keratin makes peptide bonds more accessible for digestion by the secreted proteases. Cooperation between the proteases and reducing agent results in the formation of smaller peptides and amino acids that can be taken up by the fungal cell. Cysteine, created during the decomposition of keratin, is toxic, but can be metabolised to sulphite by the action of the enzyme cysteine dioxygenase type 1 (encoded by *CDO1*), and the sulphite formed is excreted by the fungal cell again, which further facilitates keratin degradation^3,4^. Thus, the ability to degrade keratin is one of the important virulence attributes of these pathogens.

Metabolomics, following transcriptomics and proteomics, plays an important role in the understanding of the mechanisms of bacterial^5–7^, fungal^8,9^ or viral^10,11^ pathogenesis and becoming very useful in life sciences. Compared to the genome and transcriptome, the proteome and metabolome are much more complex and dynamic^12,13^. The gene expression does not always reflect directly in the amount and activity of compounds such as proteins and metabolites in the cells^14^. However, by comparing profiles of metabolites or proteins of control samples with samples growing under specific conditions, information about qualitative and quantitative changes, induced by the stimuli factor, is obtained^15^. Mass spectrometry (MS) is the basic omics method of choice in case of metabolomic studies^8^. This analytical tool is very useful for a better understanding of the biology of organisms and their response to different environmental stimuli^16^. Cellular processes are characterised by the production of unique, chemical “fingerprints”, a set of metabolites with specific compositions^17^. Thus, in the present study, liquid chromatography with tandem mass spectrometry (LC–MS/MS) based, untargeted metabolomic analysis of *T. rubrum* and *M. canis* cells, was performed. The cells grew in the presence of a two carbon sources such as keratin, which mimic the dermatophyte infection to host cells and glucose. Determination of characteristic metabolites of these two dermatophytes originated from different niches may expand knowledge about their pathogenic properties.

## Results

### Keratinolytic activity of *T. rubrum* and *M. canis* and the pH changes

**Figure 1:**
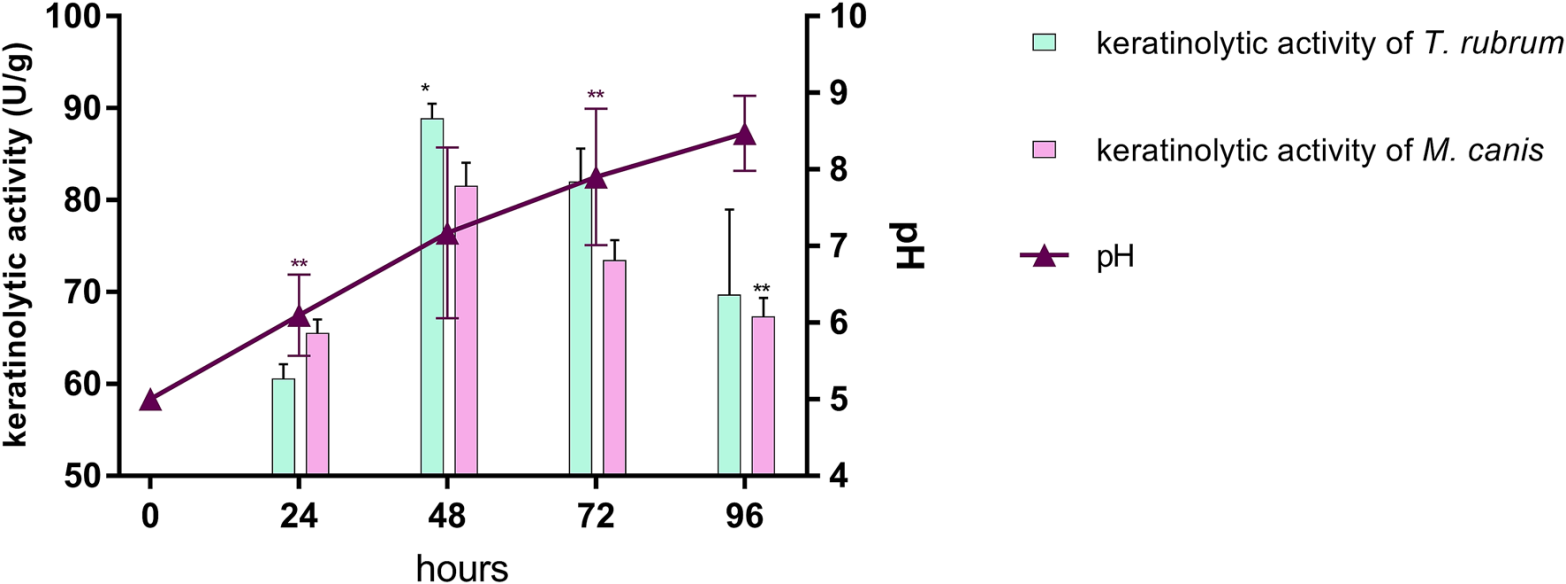
Evaluation of keratinolytic activity and pH changes of *T. rubrum* and *M. canis* at 30 °C in the presence of keratin. Keratinolyctic activity was calculated in unities/g (U/g). The bars in the columns and triangles represents the standard deviation of data obtained from three independent replicates. Significant statistical differences are represented by asterisks (*p < 0.5; **p < 0.01).

The obtained results revealed that *T. rubrum* and *M. canis* isolates have ability to degraded keratin. The production of keratinolytic enzyme was found at its maximum of 88.9 U/g in case of the culture filtrate of *T. rubrum* and 81.6 U/g in case of the culture filtrate of *M. canis* (Fig. 1) after 48 h of incubation. The initial pH of the keratin medium during growth of *T. rubrum* and *M. canis* increased from 5.0 to 8.7 after 24–96 h of incubation (Fig. 1). The differences between these averages was statistically significant (*p* < 0.05).

### Metabolomic analysis of *T. rubrum* and *M. canis*

**Figure 2:**
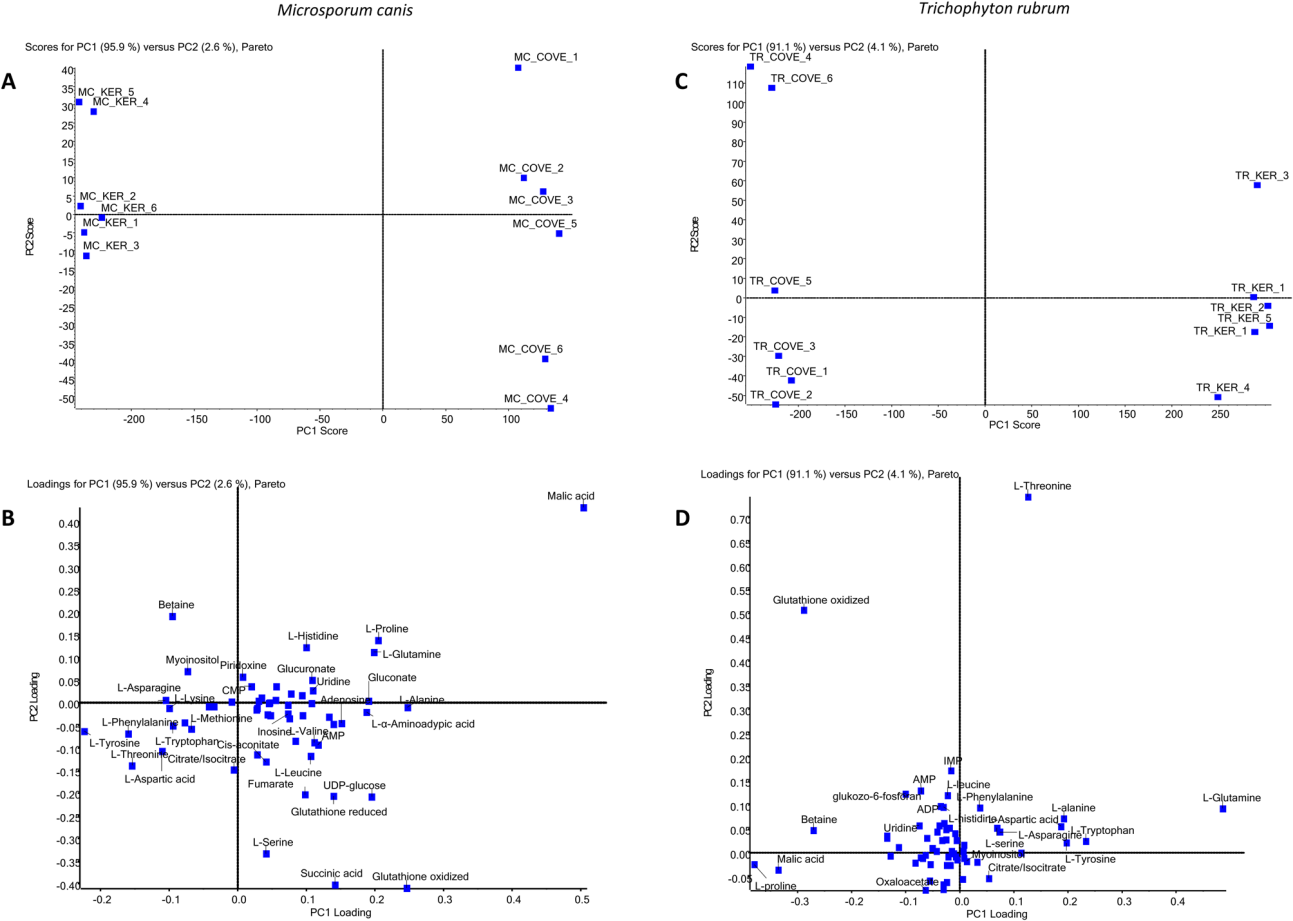
(**A**,**C**) PCA of the intracellular metabolites in minimal medium supplemented with glucose (TR-COVE, MC-COVE) or keratin (TR-KER, MC-KER) as a carbon sources (**B**,**D**) 2D PCA scores plots indicate statistically significant sample separation along the first dimension (PC1).

Quantitative analysis of intracellular metabolites of *T. rubrum* and *M. canis* obtained by separation using the LC–MS/MS method, was performed using PCA and conditional formatting in Microsoft Excel. A total of 90 intracellular metabolites (Table S1) from *T. rubrum* and *M. canis* were measured after 48 h incubation in minimal medium (MM-Cove) supplemented with glucose or keratin as a carbon sources, across the three biological and technical replicates. A 62 and 58 compounds were identified in *T. rubrum* and *M. canis* respectively, and noticeable interspecific differences between the metabolites, were observed. The PCA detected metabolites presenting statistically significant differences, between the two experimental conditions (Fig. 2A,C). Figure 2B,D presents the distribution of individual metabolites in 2D PCA scores plots. In case of *T. rubrum*, detected metabolites, such as l-proline, betaine, and malic acid with spectra on the left side of the graph, presented higher concentrations in the control medium, while intracellular metabolites, such as l-glutamine, l-tryptophan, and l-tyrosine, with spectra located on the right side of the graph, presented higher concentrations in the medium supplemented with keratin (Fig. 2D). In case of *M. canis*, detected metabolites such as asparagine, l-tyrosine, l-threonine or l-aspartic acid with spectra on the left side of the graph presented higher concentrations in the medium supplemented with keratin. However, metabolites such as l-alanine, l-proline, and reduced and oxidised glutathione with spectra on the right side of the graph, presented higher concentrations in the control medium (Fig. 2B). Other metabolites such as histidine, l-leucine, and l-phenylalanine in case of *T. rubrum* as well as isocitric acid and cytidine-5′-monophosphate in case of *M. canis*, whose spectra were in the centre of the graph, showed similar concentration ratios for the control medium, and the medium supplemented with keratin (Fig. 2B,C).

**Figure 3:**
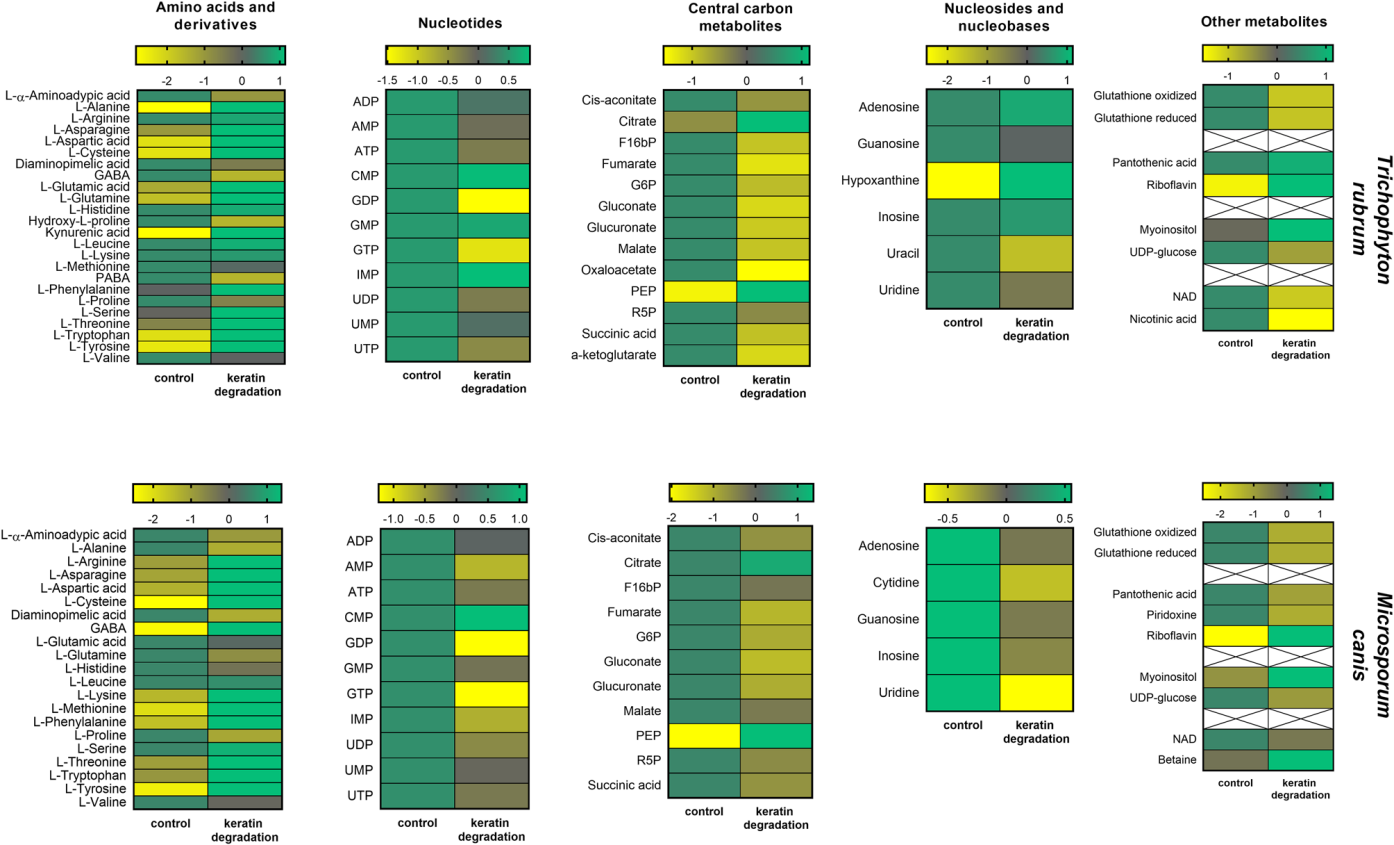
Heat map visualisation for all detected intracellular metabolites of *T. rubrum* and *M. canis* in control medium and during keratin degradation.

In case of *T. rubrum*, among 62 intracellular metabolites (Fig. 3), 45 (73%) were marked as being produced more intensively in the control medium, seven of them, such as hydroxy-l-proline, nicotinic acid, oxaloacetate, uracil, γ-aminobutyric acid (GABA), *p*-aminobenzoic acid (PABA), and guanosine-5′-diphosphate (GDP) were detected only in the control medium. In turn, 17 compounds (27%) were detected as being produced more intensively in the medium supplemented with keratin, and three out of these, l-alanine, kynurenic acid and cysteine were present only in this growth environment. Two of the three vitamins found, namely nicotinic acid and PABA, were classified as specific to the control medium, while the third, riboflavin is produced more intensively in the presence of keratin. Increased synthesis of amino acids such as l-alanine, l-tyrosine, aspartic acid, l-tryptophan, and glutamine was observed when the fungus degraded keratin present in the medium (Fig. 3).

Among the 58 identified intracellular metabolites in *M. canis* (Fig. 3), 42 compounds (72%) were up-regulated in the control medium, while 16 metabolites presented higher concentration values in the medium supplemented with keratin. Six compounds namely l-alanine, diaminopimelic acid (DAP), reduced glutathione, oxidised glutathione, guanosine-5′-triphosphate (GTP), and pyridoxine (vitamin B6) were detected only in the control medium, while two compounds (Fig. 3), cysteine and riboflavin were detected only in the medium supplemented with keratin. A reduced concentration of amino acids, TCA-and glycolysis-related metabolites, and nucleotides was observed in the medium supplemented with keratin. The fungus intensively produced, intracellular metabolites such as GABA, l-tyrosine, l-phenylalanine, l-methionine, and phosphoenolpyruvic acid (Fig. 3) in the medium supplemented with keratin.

### Validation of metabolomic results by quantitative polymerase chain reaction (qPCR) analysis

**Figure 4:**
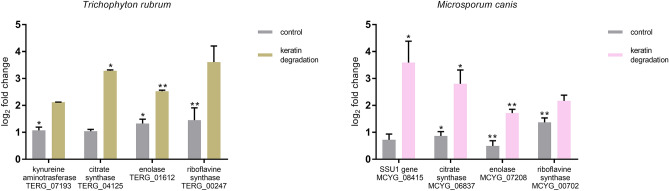
Relative gene expression analysis of selected genes involved in biosynthesis pathways of corresponding metabolites in *T. rubrum* and *M. canis*. Asterisks indicate the statistical significance as determined by Student’s t-tests (*p < 0.05; **p < 0.001).

**Figure 5:**
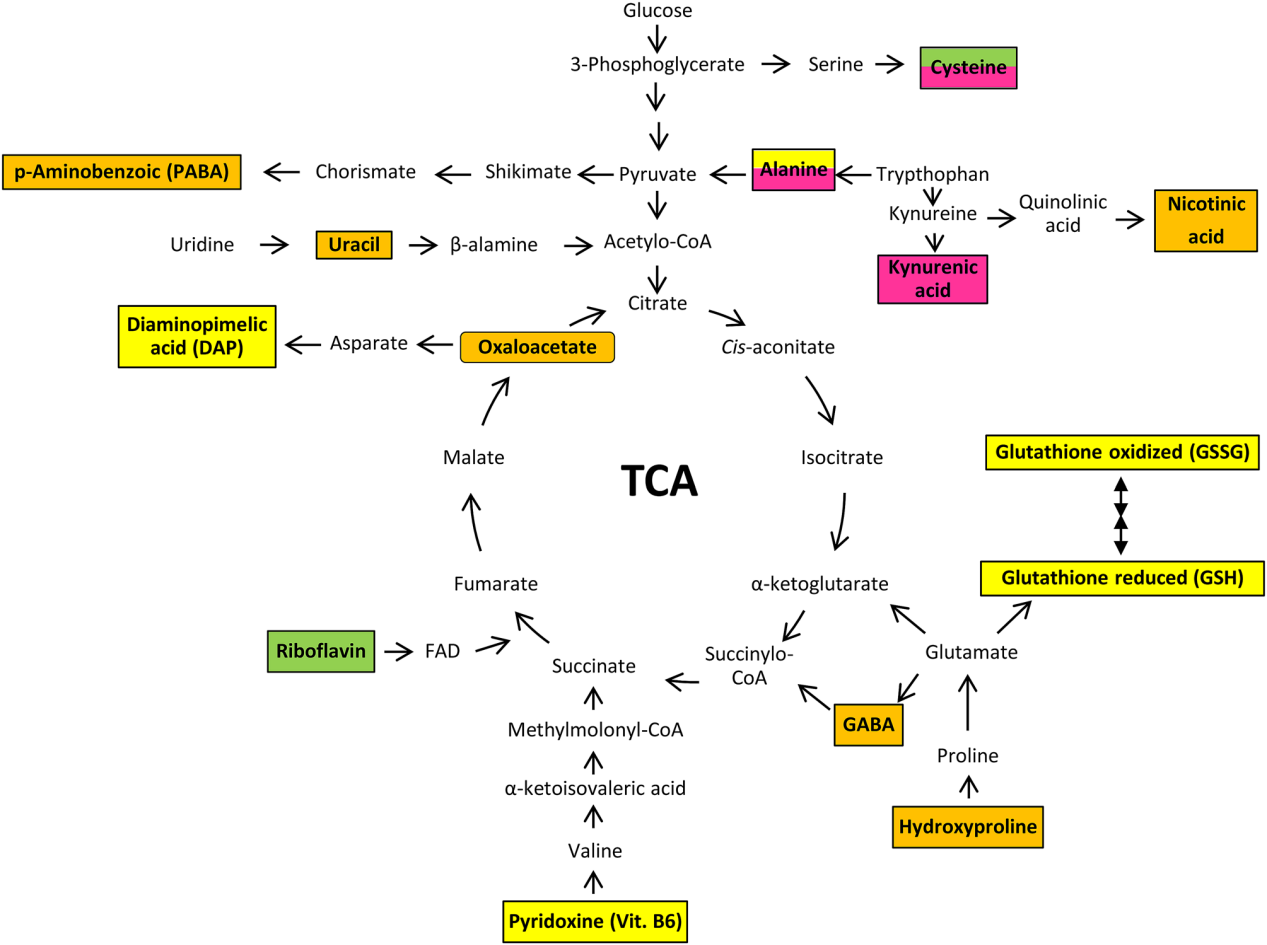
Schematic overview of the metabolomic pathway associated with the detected biochemical compounds. Metabolites of *T. rubrum* on an orange background represents those marked only in minimal medium (MM-Cove) while metabolites on a pink background represents those marked only during keratin degradation. Metabolites of *M. canis* on a yellow background represents those marked only in minimal medium (MM-Cove) while metabolites on a green background represents those marked only during keratin degradation.

The validation was performed using templates from the *T. rubrum* CBS 120358 and *M. canis* CBS 11348 strains, incubated at 28 °C for 48 h in the control medium and in the medium supplemented with keratin. Four genes for *T. rubrum* and four genes for *M. canis* (Table 1) encoding the identified metabolites (Fig. 3) were randomly selected. Kynurenine aminotransferase (TERG_07193) catalyses the synthesis of kynurenic acid in the kynurenine pathway, while citrate synthase (TERG_04125, MCYG_06837) is a key enzyme of the TCA cycle, catalysing the synthesis of citric acid from acetyl-CoA. Enolase (TERG_01612, MCYG_07208) known as phosphopyruvate hydratase is a glycolytic enzyme responsible for the catalysis of conversion of 2-phosphoglycerate to phosphoenolpyruvate (PEP). Riboflavin synthase (TERG_00247, MCYG_00702) is an enzyme that catalyses the final reaction of riboflavin synthesis. *SSU1* gene (MCYG_08415) was found to be strongly activated during the growth of dermatophyte cells in the presence of cysteine. The qPCR data (Fig. 4) and the corresponding metabolomic results (Figs. 3, 5) from the three biological replicates confirmed the reliability of the metabolomic data obtained (Pearson’s correlation, r > 0.97, *p* < 0.001).

**Table 1:** List of target genes and primer sequences for qPCR.

Accession no.	Gene product name	Primers (5′–3′) Forward Reverse	Length [bp]	Tm [°C]	Efficiency (%)	R^2^
TERG_07193	Kynurenine aminotransferase	GCAAGCAACGAGCCTTTCAA	196	60.5	101	0.99814
		GGTTAGACTCCACGGTCTGC				
TERG_04125	Citrate synthase	GGCTCCTACCCTCAAGGAGA	195	60.5	100	0.99755
		CGGAAGCGAATACCCTCCTC				
TERG_01612	Enolase	GAAGGGTGTCCCACTGTACG	132	60.5	97	0.99675
		AACTCCTGGAAAGCGAGACG				
TERG_00247	Riboflavin synthase	GGGCGGGACATCCTTAACAA	190	60.5	102	0.99855
		GACTGGTGATCCGGCTTTGA				
MCYG_08415	SSU1 gene	AAGAGCTTCAGGTCACAGCC	191	60.5	99	0.99655
		AAGCCCGGGAAACTGGTATG				
MCYG_06837	Citrate synthase	GACACAAGGTCCTCGGTGAG	146	60.5	108	0.99723
		GAGTTTCTGGCACTCGGGAA				
MCYG_07208	Enolase	CGGTGTCAGTTTGGCCATTG	171	60.5	101	0.99651
		AACTCCTGGAAGGCAAGACG				
MCYG_00702	Riboflavin synthase	AGCTCCTTTCAGTCACACCG	113	60.5	98	0.99490
		AGTGCCGTCAAGAGCAATGA				
XM_002848521	ADP ribosylation factor (*adp-rf*)	GAATTCTCATGGTCGGTCTC	104	60.5	100	0.99855
		AACGTTGAATCCGATGGTG				
XM_002843632	Multiubiquitin chain binding protein 1 (*mbp1*)	AGTCCTAGTTACCTTGACCG	123	60.5	99	0.99924
		CGGTGTTTAAGTGCTAGATAGG				
TERG_00548	Elongation factor 1-alpha (*ef1-α*)	GAGAAGTTCGAGAAGGAAGC	128	60.5	107	0.99803
		TGACGGTGACATTGTACTTG				
TERG_04033	Ribosomal protein L2 (*rpl2*)	GGATCTATATTCACGGCTCG	113	60.5	102	0.99484
		TGGATGATGTTCTTCACGAC

## Discussion

*Trichophyton rubrum* and *Microsporum canis* are group of fungi classified as anthropophilic and zoophilic, respectively, what is related to their natural habitat^1^. These two dermatophyte species differ also in host range as well as dermatophytosis progression. Moreover, both of them during infection can evade the host immune system what leads to the chronic infections^18^. Previously study about a comparative genome analysis of five dermatophytes species such as *T. rubrum*, *T. tonsurans*, *T. equinum*, *M. canis* and *M. gypseum* revealed some interspecific differences between them, what suggested that various gene as well as post-transcriptional regulation are known as the dermatophyte arsenal necessary to survive in the various niches and different hosts^19^. Extending of studies to metabolomic analysis to compare other interspecies differences are required to complete answers to the questions how these fungal pathogens adapt to different niches as well as how they utilize the available nutrients necessary for its survival and growth during the host–dermatophyte interaction.

The beginning of any dermatophyte infection in vivo is in the form of a direct contact of the pathogen cells with the target host structure. If the current condition of the host is conducive to the initiation of infection, arthroconidia of dermatophytes adhere to keratinised tissues and begin their penetration process^20^. Dermatophytes produce a broad spectrum of proteolytic enzymes which are essential to their pathogenicity as well as virulence, are the key proteins involved in keratin degradation^21^. Degradation of keratin by dermatophytes releases amino acids, whose metabolism leads to secretion of ammonia, which changes the initial pH in the host tissue from acidic (pH 5.0) to alkaline (pH 8.5) approximately within 72–96 h, producing an environment in which most of the known keratinolytic proteases of dermatophytes have optimal enzymatic activity. This in turn is extremely important for the growth of the dermatophytes in the host tissue^2,21–26^. In this work, we reported that *T. rubrum* and *M. canis* have the highest keratinolytic activity at 48 h time point. Our results demonstrated also that the optimum pH for keratinases production was slightly alkaline (pH 7.2) (Fig. 1) what agreed with the previously reported data^2,21–24^. Therefore, we decided to choose the measuring point at 48 h, to identify intracellular metabolites in *T. rubrum* and *M. canis*, during the degradation of keratin by these fungi. The keratin obtained from sheep wool, human hair or bird feathers is characterised by a high content of amino acids such as (1) non-ionic polar amino acids, for e.g., serine, cysteine, threonine, and tyrosine; (2) ionic polar amino acids, for e.g., arginine, lysine, aspartic acid, and glutamic acid; and (3) non-polar amino acids, for e.g., alanine, glycine, leucine, methionine, phenylalanine, proline, and valine^27^. All these amino acids were detected, in the present study, in *T. rubrum* as well as in *M. canis*, in the control medium, and in the medium supplemented with keratin (Figs. 3, 5). After 48 h, a significant increase in the amount of intracellular amino acids and their derivatives were observed, when *T. rubrum* and *M. canis* degraded keratin (Figs. 3, 5) which can then be used as a sources of carbon, nitrogen as well as sulphur^2,28,29^.

Compounds such as l-alanine, kynurenic acid and cysteine in case of *T. rubrum*, as well as cysteine and riboflavin in case of *M. canis* were detected only in the medium supplemented with keratin (Figs. 3, 5). The significant increase in the production of kynurenic acid by *T. rubrum* in the medium which promotes keratin degradation is interesting (Figs. 3, 4, 5). Kynurenic acid depicts strong immunosuppressive properties on vertebrate immune cells, leading to the silencing of their inflammatory action by limiting the production of cytokines, including interleukins (IL) such as IL-4, IL-23, and tumour necrosis factor-α (TNF-α). Furthermore, it inhibits Th17 lymphocyte differentiation^30^. IL-17 produced by the Th17 cells, plays an important role in activating antifungal response^31,32^. These reports seem particularly interesting, as *T. rubrum*, as an anthropophilic dermatophyte, has developed a number of strategies to avoid the host immune response, which directly affects the duration of infections caused and the difficulties in their treatment^33^. In turn, l-alanine is one of the key amino acids that constitutes proteins, and its increased intracellular presence found only when *T. rubrum* degraded keratin (Figs. 3, 5) may suggest that l-alanine is an important component of proteins responsible for the pathogenesis process of the dermatophyte fungi. In case of the highly virulent mutant of another fungal pathogen, *Candida albicans* pir32 null strain, l-alanine is part of the sequence of the most identified cell wall proteins (CWPs). Analyses conducted on other pathogenic fungi have shown that CWPs play an essential role in the biosynthesis and rearrangements of the cell wall, and are important virulence factors^34,35^. During the metabolomic analyses of *T. rubrum* and *M. canis*, cysteine was detected only during the degradation of keratin by these fungi (Figs. 3, 4, 5). Cysteine, a product of keratin degradation, is oxidised by cysteine dioxygenase (CDO) to cysteine sulphuric acid. This compound is a precursor of taurine, or pyruvate and sulphate, which can be reused in the degradation of keratin^4^. Cysteine is a very good source of sulphur and is quickly used up from the medium by the pathogen, even in the presence of better nutrients. Moreover, the excess sulphur is excreted outside the cell in the form of inorganic compounds^36^. The studies on *Trichophyton mentagrophytes* mutants, which were unable to produce cysteine dioxygenase, showed that the fungus grew very poorly on hair and nails, and was highly sensitive to cysteine^3^. Intracellular cysteine, detected in the present study in *T. rubrum* and *M. canis* only in the medium supplemented with keratin, confirmed that the pathogens degraded keratin present in the medium and then used it as a substrate for sulphite formation.

Riboflavin (vitamin B_2_) was the second intracellular metabolite that was observed in *M. canis* only during keratin degradation (Figs. 3, 4, 5). Dietl et al.^37^ showed that vitamin B_2_ is crucial for the pathogenesis of another filamentous fungi *Aspergillus fumigatus*. The study had revealed that removal of the gene involved in the process of riboflavin biosynthesis (*riboB*) directly results in loss of virulence of this pathogen^37^. Riboflavin is a precursor of flavin mononucleotide (FMN) as well as flavin adenine dinucleotide (FAD), which are cofactors required for catabolic oxidative processes. Flavoproteins produced by *A. fumigatus* are responsible for the biosynthesis of siderophores or iron assimilation^38^. The virulence of pathogens is often characterised based on the ability of these microorganisms to obtain iron from the environment^39^. Therefore, increased riboflavin production (Figs. 3, 4, 5) by *M. canis* during degradation of keratin may suggest that it is one of the adaptations to the infection process. The biochemical pathway for the synthesis of this metabolite could be a proposed target for an antifungal drug.

It was observed that the growth of *T. rubrum* and *M. canis* in the control medium (minimal medium containing glucose as the only carbon source), intensively produced the main products of the intracellular respiration process (Figs. 3, 5). Maranhẚo et al.^40^ reported that *T. rubrum* growing in the presence of glucose as the only available carbon source acidifies the medium as well as inhibit the production of external proteases. Glycolysis metabolites can be included in further stages of the process, but they can be alternatively used in other metabolic pathways. For example, the inclusion of glucose-6-phosphate in the pentose phosphate pathway results in the presence of ribose-5-phosphate and nicotinamide adenine dinucleotide phosphate (NADPH) which provides more effective cell protection in conditions of oxidative stress^41^. The minimal medium (MM-Cove) containing glucose as a carbon source used in this study^42^ due to the limited nutrient availability for *T. rubrum* as well as *M. canis* may lead to increased production of specific protective substances and mechanisms by the microorganism^43^. Moreover, the increased number of metabolites that act as energy transporters, such as GTP, adenosine triphosphate (ATP) or uridine-5′-triphosphate (UTP) in the control medium (Fig. 3) can be useful for the increased synthesis of molecules which have a protective effect on *T. rubrum* and *M. canis* cells. In this context, it is worth focusing on hydroxy-l-proline produced by *T. rubrum* only in the control medium (Figs. 3, 5). In *Saccharomyces cerevisiae* cells, l-proline and hydroxy-l-proline have been shown to have a protective function during endoplasmic reticulum (ER) stress in the presence of low level of amino acids, induced by nutrient deficiency. ER stress results in incorrect folding of produced proteins as well as reduction of total protein production in the cell. *S. cerevisiae* mutants without the ability to synthesise proline, are characterised by increased sensitivity to ER stress. Moreover, higher concentrations of compounds such as glutathione and NADPH were also recorded in these cells^44^. It is interesting to note that oxidised and reduced glutathione were detected only in the control medium, both in case of *T. rubrum* and *M. canis* (Figs. 3, 5). Glutathione play an important role in the protection against xenobiotics, heavy metals and reactive oxygen species among aerobic prokaryotes and eukaryotes^45^. Moreover, in the presence of limited nutrients in the environment, glutathione is an important reservoir of nitrogen and sulphur^45^. Previously reports indicated also that compounds such as pyridoxine (vitamin B6)^46^, the metabolite marked together with glutathione in *M. canis* only in the control medium as well as para-Aminobenzoic acid (PABA)^47,48^ and γ-aminobutyric (GABA)^49^ the metabolites marked in *T. rubrum* in the control medium may help the cell survive under stress environment such as limited nutrient availability (Fig. 3).

## Conclusion

Keratin is a compact protein, and its utilisation by dermatophytes seems to be a major virulence attribute of this group of medically important fungi. The results of this study suggest that degradation of keratin produces changes in the intracellular metabolic activity of *T. rubrum* and *M. canis*. Despite some common features in the species analysed, several characteristic molecules such as cysteine, l-alanine and kynurenic acid for *T. rubrum* and cysteine, and riboflavin for *M. canis* were detected during keratin degradation. All these compounds appear to be important in the pathogenesis process of the fungi. The effects of this metabolomic analysis of *T. rubrum* and *M. canis* during keratin degradation what mimic the dermatophyte infection to the host cells, provides an opportunity to improve the knowledge available on mechanisms associated with the pathogenicity of dermatophytes and other biological properties of this group of pathogens. The next step of our research will be the identification of proteins (proteomic analysis) and whose level increases or attenuates in response to different sources of carbon (glucose and keratin) in optimum conditions of growth, which promote the adhesion of dermatophytes to host cells.

## Materials and methods

### Chemicals and reagents

All chemicals and reagents were LC–MS grade. Keratin originated from sheep’s wool (98% purity) was purchased from PROTEINA (Łódź, Poland). Water, acetonitrile, formic acid, and ammonium acetate were purchased from Avantor (Gliwice, Poland) and Merck (Munich, Germany). Ninety metabolites were purchased from Merck (Munich, Germany) (Table S1).

### Culture conditions and extraction of intracellular metabolites

The strains *T. rubrum* CBS 120358 and *M. canis* CBS 11348 from Westerdijk Fungal Biodiversity Institute (formerly CBS-KNAW Collections), the Netherlands, were used in this study. Conidia from *T. rubrum* and *M. canis* strains (approximately 10^7^ cells/ml) were isolated as described previously by Dobrowolska and Stączek^50^ and germinated into YG medium contained 0.5% yeast extract as well as 2% glucose and cultivated for 72 h at 28 °C with agitation^51,52^. Next, the cultures were transferred and incubated separately for 48 h at 28 °C with agitation in liquid minimal medium (MM-Cove) at initial pH 5.0^42^ containing 70 mM sodium nitrate and either 50 mM glucose (control medium) or 0.5% (w/v) keratin as a carbon sources. Mycelia from three independent biological replicates of *T. rubrum* or *M. canis* cultures (with glucose or keratin) were collected at 48 h and used for metabolomic analysis. Liquid *T. rubrum* and *M. canis* cultures were filtered through a disposable filter with a 0.2 µm pore diameter and washed using 100 ml of deionised water. Next, 100 mg of the wet biomass was transferred to 2 ml LoBind tubes and frozen with liquid nitrogen. Metabolites were extracted by mechanical lysis of the entire extractant solution (biomass in 80% acetonitrile LC–MS/20% demineralised water V/V) with frozen 3 mm tungsten carbide beads in a TissueLyser II homogeniser (Qiagen, Hilden, Germany) for five cycles of 1 min each at 30 Hz speed. The lysates were clarified by centrifugation at 12,000 rpm for 5 min at 4 °C.

### Keratinolytic assay

The keratinolytic potential of *T. rubrum* and *M. canis* was performed according to previously described protocols^53–55^ with modifications. In brief, 1 × 10^7^ cells/ml of *T. rubrum* and *M. canis* were germinated into YG medium (2% w/v glucose, 0.5% w/v yeast extract) for 72 h. After the incubation time, the cultures were filtered using Falcon 40 µm Cell Strainer (Corning, New York, USA) and then transferred to the 25 ml of keratin medium (2.5 g/l) (PROTEINA, Łódź, Poland) at pH 5.0, the material was incubated for 24, 48, 72 and 96 h at 30 °C under constant agitation (120 rpm). The pH of medium was measured with Five Easy Plus pH/mv bench meter (Mettler Toledo, Columbus, USA) for each day by retrieving 2 ml liquid. Next, mycelia of *T. rubrum* and *M. canis* after 24, 48, 72 and 96 h of incubation were filtered using Falcon 40 µm Cell Strainer (Corning, New York, USA) and the 1.0 ml of supernatants as the enzymes were evaluated for keratynolytic activity. The reaction mixture contained 50 mM Tris–HCl buffer (pH 8.0), 20 mg of keratin powder (PROTEINA, Łódź, Poland) and 1 ml of fungal supernatant containing extracellular keratinase in total volume of 4 ml and was incubated for 30 min at 45 °C with shaking (160 rpm). The enzyme reaction was terminating by addition of 1 ml of 10% (w/v) trichloroacetic acid (TCA) and cooled at 4 °C for 30 min. After centrifugation (10,000 rpm, 15 min), the absorbance of the supernatants was measured by spectrophotometer (Spectra Max i3, Molecular Devices, San Jose, USA) at wavelength of 595 nm. A change of 0.01 A595 per hour equals 1 U (unit) of keratinase activity.

### LC–MS/MS conditions

Quantitative analysis of the lysates was performed using the Eksigent microLC 200 (Sciex, Framingham, Massachusetts, USA) and Agilent 1200 (Agilent, Santa Clara, California, USA) liquid chromatograph (LC) systems coupled with tandem mass spectrometer QTRAP 4500 (Sciex, Framingham, Massachusetts, USA). LC separation was carried out on three different columns in the reverse-phase mode. Vitamins, amino acids, and nucleobases were separated using a Column 1: Eksigent 3C8-EP-120 (0.5 × 150 mm, 3 μm) column (Eksigent Technologies Inc., Dublin, California, USA) in positive ionisation mode (Tables S2, S3). The mobile phases consisted of 0.1% formic acid in water (mobile phase A) and 0.1% formic acid in acetonitrile (mobile phase B). Selected organic acids, pantothenate, and both forms of glutathione (reduced and oxidised) were separated using a Column 2: Eksigent C318-AQ-120 (0.5 × 150 mm, 3 μm) column Eksigent Technologies Inc., Dublin, California, USA) in negative ionisation mode (Table S2). 0.1% formic acid in water (mobile phase A) and 0.1% formic acid in acetonitrile (mobile phase B) were used as the mobile phases (Tables S2, S3). Other organic acids, nucleotides, and molecules with phosphate groups were separated using a Column 3: Synergi Hydro-RP (2 × 150 mm, 4 μm) (Phenomenex, Torrance, California, USA) in negative ionisation mode (Tables S2, S3). The mobile phases consisted of 4 mM ammonium acetate in 95:5 acetonitrile–water (mobile phase A) and 4 mM ammonium acetate in 5:95 acetonitrile–water (mobile phase B). The detailed conditions of the LC–MS/MS analysis of this study are available in Tables S2 and S3.

### Quantitative polymerase chain reaction (qPCR) validation

The expression patterns of eight randomly selected genes encoding the identified metabolites were analysed with primer pairs (Table 1) designed with the Primer 5 program (Premier Biosoft International, California, USA). cDNA was synthesised using 2 μg of total RNA (DNA-free), RevertAid reverse transcriptase (Thermo Scientific, Waltham, MA, USA) and Random Hexamer Primers (5′-NNNNNN-3′; N = G, A, T or C) (Thermo Scientific, Waltham, MA, USA) following the manufacturer’s protocol. The qPCR assay was conducted on a Rotor-Gene Q System (Qiagen, Hilden Germany) based on a method described previously^51,52^ using SsoAdvanced Universal SYBR Green Supermix (2×) (Bio-Rad, Hercules, California, USA). The mixtures were subjected to an initial step at 95 °C for 1 min, followed by 40 cycles of denaturation at 95 °C for 20 s, annealing at 60.5 °C for 20 s, and elongation at 72 °C for 15 s. Melting curve analysis was performed by heating the amplicon from 72 °C to 95 °C. Relative gene expression levels were calculated according to the 2-ΔΔCT method, with *ef1-α* and *rpl2* for *T. rubrum*, and *adp-rf* and *mbp-1* for *M. canis* (Table 1) as the reference genes according to the MIQE (Minimum Information for Publication of Quantitative Real-Time PCR Experiments) guidelines^56^.

### Statistical analysis

All experiments were carried out in biological and technical triplicate. Pearson's rank correlation analysis was conducted to calculate the correlation between metabolites or between species and metabolites. Differences were considered significant when *p* < 0.05. After the data matrix was mean-centred and scaled to the Pareto variance, principal component analysis (PCA) was conducted with MarkerView software (Sciex, Framingham, Massachusetts, USA). The quantitative data on intracellular metabolites of *T. rubrum* and *M. canis* obtained by separation through LC–MS/MS were normalised using the Z-score algorithm on Microsoft Excel (Microsoft Corporation, Seattle, WA, USA) and heat maps were created using the GraphPad Prism 6 software (GraphPad software Inc., San Diego, California, USA).

## Supplementary Information

Supplementary Tables.
