# Supplementary material for: Metabolomic analysis of Trichophyton rubrum and Microsporum canis during keratin degradation
Source: Sci Rep. 2021 Feb 17;11:3959. doi: 10.1038/s41598-021-83632-z (PMC7889620; doi:10.1038/s41598-021-83632-z)
Supplement: Supplementary file 1 — Supplementary Tables. [file 41598_2021_83632_MOESM1_ESM.pdf]

SUPPLEMENTARY MATERIALS

Metabolomic analysis of *Trichophyton rubrum* and *Microsporum canis* during keratin degradation

Anita CIESIELSKA[1], Anna KAWA[1], Katarzyna KANAREK[1], Adrian SOBOŃ[1], Rafał SZEWCZYK[2]

[1] Department of Molecular Microbiology, Faculty of Biology and Environmental Protection, University of Łódź, Poland

[2] LabExperts sp. z o.o., Gdańsk, Poland

Tables:

Table S1 List of metabolites used in this study

Table S2 LC-MS/MS parameters used for analysis of intracellular metabolites of *T. rubrum* and *M. canis*

Table S3 MS/MS parameters and LC-MS/MS method performance for compound standards

Table S1 List of metabolites used in this study

**1**	5-aminovalerate	**21**	FAD	**41**	Inosine	**61**	L-α-Aminoadipic acid	**81**	Tartrate
**2**	Acetyl-CoA	**22**	Fumarate	**42**	Kynurenic acid	**62**	Malate	**82**	Thiamine
**3**	Adenine	**23**	G6P	**43**	L-Alanine	**63**	Malonate	**83**	Tocopherol
**4**	Adenosine	**24**	GABA	**44**	L-Arginine	**64**	Melatonin	**84**	UDP
**5**	Adipate	**25**	GDP	**45**	L-Asparagine	**65**	Myoinositol	**85**	UDP-glucose
**6**	ADP	**26**	Gluconate	**46**	L-Aspartic acid	**66**	NAD	**86**	UMP
**7**	AMP	**27**	Glucuronate	**47**	L-Cysteine	**67**	NADP	**87**	Uracil
**8**	ATP	**28**	Glutathione oxidized	**48**	L-Glutamic acid	**68**	Nicotinic acid	**88**	Uridine
**9**	Betaine	**29**	Glutathione reduced	**49**	L-Glutamine	**69**	Ornithine	**89**	UTP
**10**	Biotin	**30**	GMP	**50**	L-Histidine	**70**	Oxaloacetate	**90**	α-ketoglutarate
**11**	CDP	**31**	GTP	**51**	L-Leucine	**71**	P5P		
**12**	Cis-aconitate	**32**	Guanidine acetate	**52**	L-Lysine	**72**	PABA		
**13**	Citrate	**33**	Guanosine	**53**	L-Methionine	**73**	Pantothenic acid		
**14**	CMP	**34**	Homocysteine	**54**	L-Phenylalanine	**74**	PEP		
**15**	CTP	**35**	Homocysteine thiolactone	**55**	L-Proline	**75**	Pyridoxine		
**16**	Cyanocobalamin	**36**	Homocysteine	**56**	L-Serine	**76**	Quinate		
**17**	Cystine	**37**	Homoserine	**57**	L-Threonine	**77**	R5P		
**18**	Cytidine	**38**	Hydroxy-L-proline	**58**	L-Tryptophan	**78**	Riboflavin		
**19**	Diaminopimelic acid	**39**	Hypoxanthine	**59**	L-Tyrosine	**79**	Succinic acid		
**20**	F16bP	**40**	IMP	**60**	L-Valine	**80**	Succinyl-CoA		

Table S2 LC-MS/MS parameters used for analysis of intracellular metabolites of *T. rubrum* and *M. canis*

LC parameters			
	**Column 1:** Eksigent 3C8-EP-120	**Column 2:** Eksigent C318-AQ-120	**Column 3:** Synergi Hydro-RP
**Injection volume**	2 µl	2 µl	10 µl
**Column temperature**	45°C	40°C	40°C
**MS/MS parameters**			
**Polarity**	positive	negative	negative
**CUR (Curtain gas)**	25	25	30
**CAD (Collision gas)**	medium	medium	medium
**IS (Ion spray voltage)**	5000 V	-4500 V	-4500 V
**TEM (Source temperature)**	400°C	400°C	500°C
**GS1 (Nebulizer gas)**	28	28	50
**GS2 (Drying gas)**	40	40	40
**ihe**	ON	ON	ON

GRADIENT ANF FLOW OF THE MOBILE PHASES

**step**	running time [min]	flow [µl/min]	mobile phase A [%]	mobile phase B [%]
Column 1				
**1**	0	50	98	2
**2**	0.2	50	98	2
**3**	2.2	50	5	95
**4**	3.4	50	5	95
**5**	3.5	50	98	2
**6**	4	50	98	2
Column 2				
**1**	0	50	98	2
**2**	0.1	50	98	2
**3**	0.5	50	70	30
**4**	2.5	50	2	98
**5**	3.5	50	2	98
**6**	4	50	98	2
Column 3				
**1**	0	600	100	0
**2**	0.2	600	100	0
**3**	5	600	5	95
**4**	7	600	5	95
**5**	7.1	600	100	0
**6**	15	600	100	0

Table S3 MS/MS parameters and LC-MS/MS method performance for compound standards

Metabolite Name	Q1	Q3	DP	EP	CE	CXP	RT (min)	Linearity Range (ng/ml)[1]	R	Column*
**L-α-Aminoadypic acid_1**	162.0	98.1	51	10	25	8	0.42	0.5-100	0.9996	1
**L-α-Aminoadypic acid_2**	162.0	127.1	51	10	21	8	0.42			1
**L-α-Aminoadypic acid_3**	162.0	144.1	51	10	21	6	0.42			1
**Adenosine_1**	268.0	136.0	66	10	35	10	0.55	0.25-100	0.9960	1
**Adenosine_2**	268.0	131.2	66	10	21	6	0.55			1
**Adenosine_3**	268.0	119.1	66	10	35	4	0.55			1
**ADP_1**	426.0	78.8	-95	-10	-23	-11	0.54	10-1000	0.9916	3
**ADP_2**	426.0	95.0	-95	-10	-13	-9	0.54			3
**ADP_3**	426.0	158.6	-95	-10	-29	-9	0.54			3
**L-Alanine_1**	90.1	44.0	31	10	19	6	0.39	25-250	0.9974	1
**L-Alanine_2**	90.1	49.0	31	10	21	5	0.39			1
**L-Alanine_3**	90.1	45.0	31	10	31	6	0.39			1
**AMP_1**	345.9	78.8	-100	-10	-21	-13	0.64	5-1000	0.9946	3
**AMP_2**	345.9	79.0	-100	-10	-47	-21	0.64			3
**AMP_3**	345.9	96.8	-100	-10	-14	-29	0.64			3
**L-Arginine_1**	175.1	70.2	46	10	13	8	0.34	1-100	0.9988	1
**L-Arginine_2**	175.1	70.1	46	10	19	12	0.34			1
**L-Arginine_3**	175.1	60.1	46	10	25	6	0.34			1
**L-Asparagine_1**	133.1	87.0	46	10	15	8	0.39	2.5-100	0.9951	1
**L-Asparagine_2**	133.1	85.0	46	10	39	6	0.39			1
**L-Asparagine_3**	133.1	74.1	46	10	15	4	0.39			1
**L-Aspartic acid_1**	134.1	88.1	51	10	39	12	0.39	2.5-100	0.9972	1
**L-Aspartic acid_2**	134.1	81.1	51	10	13	8	0.39			1
**L-Aspartic acid_3**	134.1	74.0	51	10	21	6	0.39			1

**ATP_1**	506.0	158.7	-15	-10	-15	-5	0.51	25-1000	0.9928	3
**ATP_2**	506.0	102.1	-15	-10	-27	-10	0.51			3
**ATP_3**	506.0	78.8	-15	-10	-17	-21	0.51			3
**Betaine_1**	118.0	58.0	76	10	41	16	0.41	1-50	0.9972	1
**Betaine_2**	118.0	50.1	76	10	21	12	0.41			1
**Betaine_3**	118.0	59.1	76	10	55	4	0.41			1
**Cis-aconitate_1**	172.8	85.0	-20	-10	-15	-5	0.95	2.5-1000	0.9995	2
**Cis-aconitate_2**	172.8	98.1	-20	-10	-15	-8	0.95			2
**Cis-aconitate_3**	172.8	129.0	-20	-10	-33	-7	0.95			2
**Citrate_1**	190.8	84.9	-15	-10	-35	-9	0.48	5-250	0.9889	3
**Citrate_2**	190.8	75.2	-15	-10	-19	-4	0.48			3
**Citrate_3**	190.8	86.9	-15	-10	-23	-7	0.48			3
**CMP_1**	321.9	78.8	-70	-10	-17	-1	0.51	10-1000	0.9965	3
**CMP_2**	321.9	100.2	-70	-10	-26	-4	0.51			3
**CMP_3**	321.9	96.9	-70	-10	-19	-7	0.51			3
**L-Cysteine_1**	122.0	59.1	61	10	27	6	0.4	1-100	0.9974	1
**L-Cysteine_2**	122.0	73.9	61	10	25	6	0.4			1
**L-Cysteine_3**	122.0	76.1	61	10	21	6	0.4			1
**Cytidine_1**	244.0	112.1	16	10	15	6	0.39	0.25-100	0.9963	1
**Cytidine_2**	244.0	96.1	16	10	39	12	0.39			1
**Cytidine_3**	244.0	94.9	16	10	27	12	0.39			1
**Diaminopimelic acid_1**	191.1	128.1	61	10	13	12	0.36	1-100	0.9998	1
**Diaminopimelic acid_2**	191.1	78.3	61	10	47	17	0.36			1
**Diaminopimelic acid_3**	191.1	82.0	61	10	21	6	0.36			1
**F16bP_1**	338.9	78.8	-15	-10	-15	-5	0.47	5-1000	0.9916	3
**F16bP_2**	338.9	96.5	-15	-10	-19	-19	0.47			3
**F16bP_3**	338.9	96.8	-15	-10	-39	-7	0.47			3

**Fumarate_1**	114.9	70.9	-35	-10	-31	-5	0.88	1-100	0.9970	2
**Fumarate_2**	114.9	96.8	-35	-10	-37	-17	0.88			2
**Fumarate_3**	114.9	59.1	-35	-10	-16	-5	0.88			2
**G6P_1**	258.9	96.8	-70	-10	-26	-7	0.5	5-1000	0.9986	3
**G6P_2**	258.9	88.1	-70	-10	-19	-17	0.5			3
**G6P_3**	258.9	78.8	-70	-10	-27	-5	0.5			3
**GABA_1**	104.1	86.0	30	4	39	5	0.37	5-250	0.9977	1
**GABA_2**	104.1	158.7	30	4	15	9	0.37			1
**GABA_3**	104.1	69.0	30	4	13	6	0.37			1
**GDP_1**	441.9	78.9	-90	-10	-27	-7	0.54	10-1000	0.9944	3
**GDP_2**	441.9	59.1	-90	-10	-43	-5	0.54			3
**GDP_3**	441.9	158.8	-90	-10	-15	-9	0.54			3
**Gluconate_1**	194.9	128.9	-65	-10	-23	-9	0.49	2.5-1000	0.9931	2
**Gluconate_2**	194.9	86.9	-65	-10	-27	-9	0.49			2
**Gluconate_3**	194.9	74.9	-65	-10	-67	-7	0.49			2
**Glucuronate_1**	192.8	112.9	-50	-10	-23	-7	0.49	2.5-1000	0.9971	2
**Glucuronate_2**	192.8	94.9	-50	-10	-19	-6	0.49			2
**Glucuronate_3**	192.8	73.0	-50	-10	-30	-5	0.49			2
**L-Glutamic acid_1**	148.0	84.0	46	10	45	6	0.4	1-100	0.9964	1
**L-Glutamic acid_2**	148.0	78.8	46	10	15	7	0.4			1
**L-Glutamic acid_3**	148.0	102.0	46	10	39	6	0.4			1
**L-Glutamine_1**	147.1	84.1	36	10	29	4	0.4	1-100	0.9924	1
**L-Glutamine_2**	147.1	101.1	36	10	24	2	0.4			1
**L-Glutamine_3**	147.1	101.1	36	10	19	8	0.4			1
**Glutathione oxidized_1**	611.1	305.9	-105	-10	-57	-11	0.86	1-250	0.9894	2
**Glutathione oxidized_2**	611.1	78.7	-105	-10	-17	-12	0.86			2
**Glutathione oxidized_3**	611.1	271.8	-105	-10	-39	-9	0.86			2

**Glutathione reduced_1**	305.9	142.8	-60	-10	-17	-11	0.75	2.5-1000	0.9966	2
**Glutathione reduced_2**	305.9	111.2	-60	-10	-55	-12	0.75			2
**Glutathione reduced_3**	305.9	271.9	-60	-10	-15	-13	0.75			2
**GMP_1**	361.9	78.8	-15	-10	-25	-11	0.55	5-1000	0.9947	3
**GMP_2**	361.9	96.0	-15	-10	-92	-10	0.55			3
**GMP_3**	361.9	96.9	-15	-10	-50	-7	0.55			3
**GTP_1**	521.9	78.9	-80	-10	-44	-13	0.48	25-1000	0.9973	3
**GTP_2**	521.9	57.1	-80	-10	-44	-12	0.48			3
**GTP_3**	521.9	423.9	-80	-10	-29	-13	0.48			3
**Guanosine_1**	283.9	152.1	6	10	19	8	0.66	0.1-100	0.9998	1
**Guanosine_2**	283.9	158.7	6	10	21	9	0.66			1
**Guanosine_3**	283.9	135.0	6	10	39	8	0.66			1
**L-Histidine_1**	156.0	110.0	61	10	27	14	0.33	2.5-100	0.9956	1
**L-Histidine_2**	156.0	78.9	61	10	50	12	0.33			1
**L-Histidine_3**	156.0	83.1	61	10	35	12	0.33			1
**Hydroxy-L-proline_1**	132.0	86.0	1	10	19	8	0.4	2.5-100	0.9947	1
**Hydroxy-L-proline_2**	132.0	73.0	1	10	25	4	0.4			1
**Hydroxy-L-proline_3**	132.0	68.0	1	10	21	4	0.4			1
**Hypoxanthine_1**	136.9	110.1	31	10	21	8	0.59	0.5-100	0.9992	1
**Hypoxanthine_2**	136.9	100.8	31	10	35	5	0.59			1
**Hypoxanthine_3**	136.9	94.0	31	10	21	6	0.59			1
**IMP_1**	347.1	347.0	-10	-10	-35	-13	0.58	25-1000	0.9847	3
**IMP_2**	347.1	57.1	-10	-10	23	-11	0.58			3
**IMP_3**	347.1	78.9	-10	-10	13	-7	0.58			3
**Inosine_1**	268.9	137.0	101	10	29	12	0.62	0.25-100	0.9981	1
**Inosine_2**	268.9	78.8	101	10	19	9	0.62			1
**Inosine_3**	268.9	119.1	101	10	21	8	0.62			1

**L-Leucine_1**	132.1	85.9	51	10	31	10	0.52	2.5-100	0.9962	1
**L-Leucine_2**	132.1	158.7	51	10	21	10	0.52			1
**L-Leucine_3**	132.1	69.3	51	10	47	8	0.52			1
**Kynurenic acid_1**	190.1	144.0	55	8	14	5	1.3	0.1-100	0.9992	1
**Kynurenic acid_2**	190.1	70.0	55	8	13	7	1.3			1
**Kynurenic acid_3**	190.1	89.0	55	8	19	7	1.3			1
**L-Lysine_1**	147.2	84.2	36	10	25	6	0.33	2.5-100	0.9979	1
**L-Lysine_2**	147.2	113.0	36	10	15	7	0.33			1
**L-Lysine_3**	147.2	67.0	36	10	39	10	0.33			1
**Malate_1**	132.9	114.9	-15	-10	15	-9	0.65	5-1000	0.9972	2
**Malate_2**	132.9	70.0	-15	-10	39	-4	0.65			2
**Malate_3**	132.9	70.9	-15	-10	13	-5	0.65			2
**L-Methionine_1**	150.1	104.1	51	10	21	8	0.47	1-100	0.9981	1
**L-Methionine_2**	150.1	96.0	51	10	15	8	0.47			1
**L-Methionine_3**	150.1	133.1	51	10	27	6	0.47			1
**Myoinositol_1**	178.8	86.9	-80	-10	17	-7	0.48	5-1000	0.9971	2
**Myoinositol_2**	178.8	96.9	-80	-10	41	-7	0.48			2
**Myoinositol_3**	178.8	71.0	-80	-10	21	-5	0.48			2
**NAD_1**	662.0	540.0	-75	-10	55	-7	1.39	2.5-1000	0.9968	3
**NAD_2**	662.0	323.0	-75	-10	15	-7	1.39			3
**NAD_3**	662.0	78.9	-75	-10	15	-7	1.39			3
**Nicotinic acid_1**	124.0	79.9	26	10	33	8	0.49	1-100	0.9990	1
**Nicotinic acid_2**	124.0	78.9	26	10	35	11	0.49			1
**Nicotinic acid_3**	124.0	53.0	26	10	19	14	0.49			1
**Oxaloacetate_1**	131.0	86.9	-55	-10	23	-5	0.5	50-500	0.9966	3
**Oxaloacetate_2**	131.0	91.0	-55	-10	17	-10	0.5			3
**Oxaloacetate_3**	131.0	112.9	-55	-10	26	-7	0.5			3

**PABA_1**	138.0	77.0	56	10	19	10	1.16	0.1-100	0.9999	1
**PABA_2**	138.0	137.0	56	10	27	6	1.16			1
**PABA_3**	138.0	94.0	56	10	19	8	1.16			1
**Pantothenic acid_1**	217.9	87.8	-70	-10	-18	-7	1	0.5-100	0.9980	2
**Pantothenic acid_2**	217.9	165.0	-70	-10	-27	-7	1			2
**Pantothenic acid_3**	217.9	146.0	-70	-10	-22	-7	1			2
**PEP_1**	166.8	78.8	-40	-10	-44	-7	0.48	2.5-1000	0.9992	3
**PEP_2**	166.8	122.1	-40	-10	-52	-9	0.48			3
**PEP_3**	166.8	62.8	-40	-10	-90	-3	0.48			3
**L-Phenylalanine_1**	166.1	120.1	26	10	17	10	0.64	1-100	0.9988	1
**L-Phenylalanine_2**	166.1	143.9	26	10	22	11	0.64			1
**L-Phenylalanine_3**	166.1	103.0	26	10	39	10	0.64			1
**Piridoxine_1**	170.0	152.1	36	10	19	6	0.43	0.5-25	0.9963	1
**Piridoxine_2**	170.0	78.9	36	10	45	8	0.43			1
**Piridoxine_3**	170.0	134.0	36	10	27	4	0.43			1
**L-Proline_1**	116.0	70.1	36	10	19	8	0.42	1-100	0.9981	1
**L-Proline_2**	116.0	86.9	36	10	53	5	0.42			1
**L-Proline_3**	116.0	68.0	36	10	39	4	0.42			1
**R5P_1**	228.9	96.9	-65	-10	-18	-5	0.5	2.5-1000	0.9944	3
**R5P_2**	228.9	86.9	-65	-10	-22	-9	0.5			3
**R5P_3**	228.9	78.9	-65	-10	-52	-11	0.5			3
**Riboflavin_1**	377.1	243.0	135	10	44	11	1.15	0.25-250	0.9997	1
**Riboflavin_2**	377.1	382.4	135	10	22	10	1.15			1
**Riboflavin_3**	377.1	172.1	135	10	44	11	1.15			1
**L-Serine_1**	106.0	60.1	1	10	15	4	0.39	5-100	0.9968	1
**L-Serine_2**	106.0	71.0	1	10	67	7	0.39			1
**L-Serine_3**	106.0	42.0	1	10	33	10	0.39			1

**Succinic acid_1**	116.8	73.0	-35	-10	-16	-5	0.89	5-500	0.9927	2
**Succinic acid_2**	116.8	78.8	-35	-10	-43	-7	0.89			2
**Succinic acid_3**	116.8	98.9	-35	-10	-14	-9	0.89			2
**L-Threonine_1**	265.0	122.1	36	10	25	4	0.32	0.25-100	0.9979	1
**L-Threonine_2**	265.0	143.9	36	10	21	12	0.32			1
**L-Threonine_3**	265.0	57.1	36	10	25	6	0.32			1
**L-Tryptophan_1**	205.1	188.0	41	10	15	8	0.98	1-100	0.9999	1
**L-Tryptophan_2**	205.1	86.9	41	10	34	9	0.98			1
**L-Tryptophan_3**	205.1	146.1	41	10	25	10	0.98			1
**L-Tyrosine_1**	182.0	165.0	36	10	15	6	0.57	0.5-100	0.9993	1
**L-Tyrosine_2**	182.0	112.1	36	10	76	7	0.57			1
**L-Tyrosine_3**	182.0	91.0	36	10	39	6	0.57			1
**UDP_1**	403.0	78.7	-60	-10	-100	-5	0.51	10-1000	0.9981	3
**UDP_2**	403.0	128.1	-60	-10	-67	-9	0.51			3
**UDP_3**	403.0	158.7	-60	-10	-32	-5	0.51			3
**UDP-glucose_1**	565.0	323.0	-90	-10	-32	-11	0.52	2.5-250	0.9974	3
**UDP-glucose_2**	565.0	437.0	-90	-10	-11	-13	0.52			3
**UDP-glucose_3**	565.0	78.9	-90	-10	-112	-7	0.52			3
**UMP_1**	322.8	78.9	-90	-10	-100	-7	0.51	10-1000	0.9973	3
**UMP_2**	322.8	96.8	-90	-10	-98	-5	0.51			3
**UMP_3**	322.8	96.9	-90	-10	-26	-7	0.51			3
**Uracil_1**	113.0	96.0	41	10	23	6	0.55	5-100	0.9990	1
**Uracil_2**	113.0	128.9	41	10	43	11	0.55			1
**Uracil_3**	113.0	70.0	41	10	19	16	0.55			1
**Uridine_1**	244.9	113.0	31	10	30	10	0.56	0.5-100	0.9988	1
**Uridine_2**	244.9	70.9	31	10	13	9	0.56			1
**Uridine_3**	244.9	70.0	31	10	45	6	0.56			1

**UTP_1**	482.9	158.7	-85	-10	-34	-5	0.5	10-1000	0.9954	3
**UTP_2**	482.9	96.8	-85	-10	-55	-8	0.5			3
**UTP_3**	482.9	78.8	-85	-10	-102	-5	0.5			3
**L-Valine_1**	118.1	57.1	66	10	39	14	0.44	5-250	0.9946	1
**L-Valine_2**	118.1	57.1	66	10	43	9	0.44			1
**L-Valine_3**	118.1	78.9	66	10	89	11	0.44			1
**α-ketoglutarate_1**	144.9	100.8	-5	-10	-12	-3	0.49	10-250	0.9973	3
**α-ketoglutarate_2**	144.9	99.3	-5	-10	-17	-11	0.49			3
**α-ketoglutarate_3**	144.9	73.0	-5	-10	-18	-7	0.49			3

[1] First MRM transition was chosen for quantitative analysis, second was confirmatory transition.

* Column 1: Eksigent 3C8-EP-120 (0.5 × 150 mm, 3 µm, Eksigent);

Column 2: Eksigent 3C18-AQ-120 (0.5 × 150 mm, 3 µm, Eksigent);

Column 3: Synergi Hydro-RP column (2 × 150 mm, 4 µm, Phenomenex)
